# The nanomaterial toolkit for neuroengineering

**DOI:** 10.1186/s40580-016-0086-6

**Published:** 2016-10-20

**Authors:** Shreyas Shah

**Affiliations:** Physiological Communications Research Group, Nokia Bell Labs, 600 Mountain Avenue, Murray Hill, NJ 07974 USA

**Keywords:** Nanomaterials, Nanoparticles, Neuroscience, Neuroengineering, Stem cells, Regenerative medicine, Drug delivery, Optogenetics

## Abstract

There is a growing interest in developing effective tools to better probe the central nervous system (CNS), to understand how it works and to treat neural diseases, injuries and cancer. The intrinsic complexity of the CNS has made this a challenging task for decades. Yet, with the extraordinary recent advances in nanotechnology and nanoscience, there is a general consensus on the immense value and potential of nanoscale tools for engineering neural systems. In this review, an overview of specialized nanomaterials which have proven to be the most effective tools in neuroscience is provided. After a brief background on the prominent challenges in the field, a variety of organic and inorganic-based nanomaterials are described, with particular emphasis on the distinctive properties that make them versatile and highly suitable in the context of the CNS. Building on this robust nano-inspired foundation, the rational design and application of nanomaterials can enable the generation of new methodologies to greatly advance the neuroscience frontier.

## Background

The fields of biology and medicine have heavily relied on advances in technology to better understand how the human body works. These advances range from the creation of simple tools to conduct surgery (e.g. scalpel), devices to measure physiological levels (e.g. electrocardiograph) and instrumentation to image the body in real time (e.g. fMRI). Besides studying the human physiology, these types of technological advances have further enhanced our capabilities to diagnose, prevent and even treat medical ailments such as disease, cancer and traumatic injuries. In general, the continual development of precision tools has enabled scientists and clinicians to acquire a remarkable breadth of knowledge about biological systems.

**Fig. 1:**
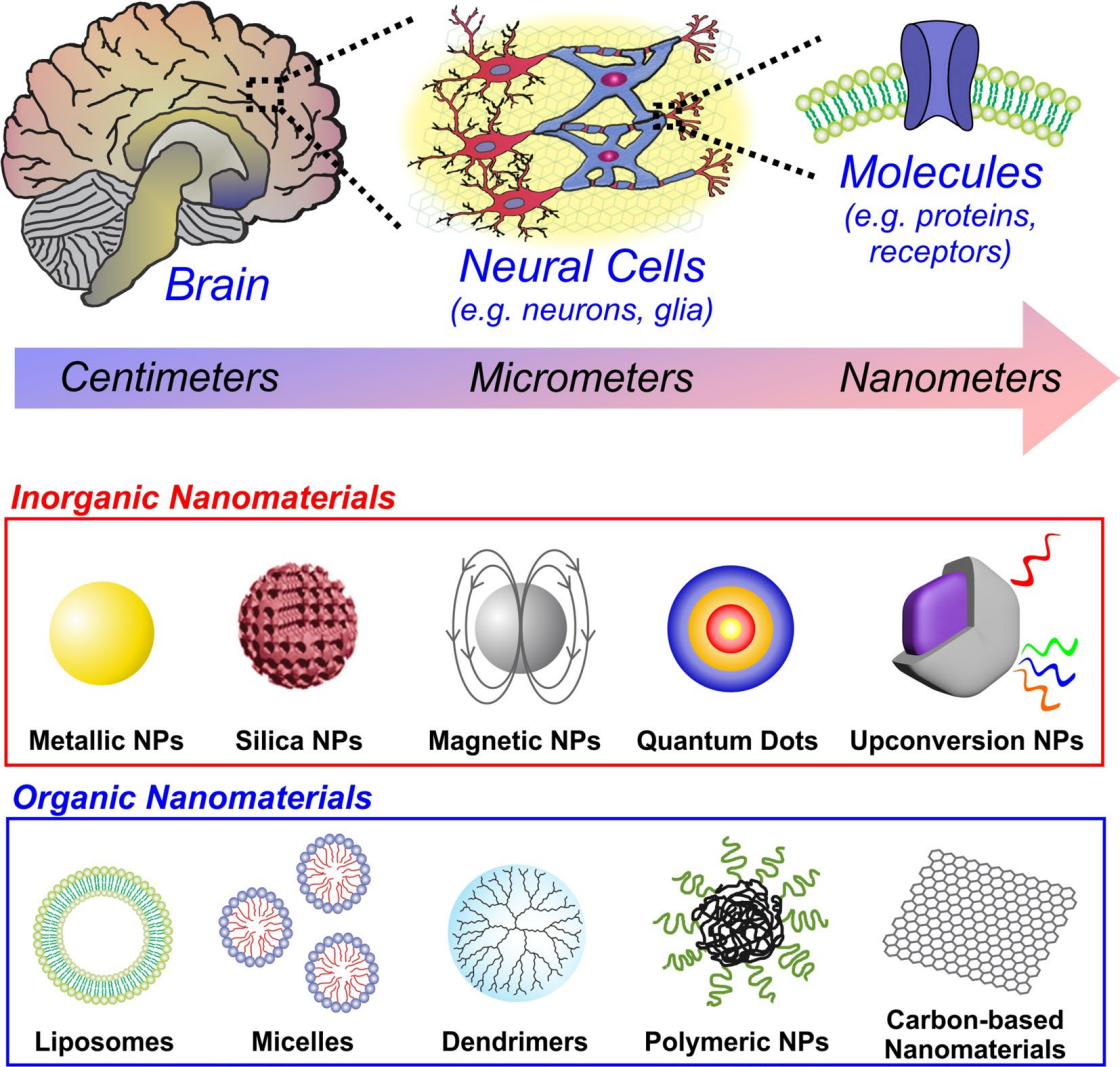
The nanomaterial toolkit for neuroengineering. Schematic depicting the anatomical organization of the brain at different size scales (*top*) and the different types of inorganic and organic nanomaterials which have been utilized for neuroscience

Among other disciplines, the field of neuroscience has greatly benefited from such advances. Neuroscientists have long strived to acquire a complete understanding of how the nervous system works. Early work involved investigating the bulk anatomical makeup of the brain, primarily through dissecting human cadavers. Taking the human brain as an example, it is organized into distinct lobes within the centimeter range (Fig. 1). The lobes were recognized to correspond to specific physiological functions, whether it be processing sensations of touch (parietal lobe) or controlling body movement (frontal lobe). Thereafter, the discovery of the role of electricity in nerve signaling, along with the development of microscopy, allowed scientists to go even deeper to the micrometer scale of neurons and glia cells. Millions of neurons in the distinct regions of the brain, are organized into ensembles or circuits, which serve to process and carry information throughout the nervous system. Going even deeper to the nanometer scale, the distinct neural cells are composed of numerous biomolecules and receptors on the surface membrane, which enable multidirectional interactions with the surrounding microenvironment. There is growing interest in the miniaturization of tools to better control and understand neural systems at this fundamental scale [1]. Biological systems function with extraordinary fidelity at the molecular level, which permits robust structure and function at the cellular, tissue and organ level. The ability to probe systems like the nervous system at the fundamental resolution that they have naturally evolved to function is revolutionary. Nanotechnology has enabled the design of materials and devices to do just that. It has enhanced our understanding of how biological systems work at the nanoscale, and further allowed the development of nanoscale tools to improve the quality of life after disease or injury [2]. The integration of engineered materials with intact biological systems has proven to be highly transformative and is possible due to the growing understanding of nanoscience and nanotechnology.

To this end, nanoscale materials hold immense potential for engineering neural systems. The ability to precisely tailor the properties of nanomaterials permits scientists and clinicians to effectively employ them for a wide variety of applications. In this review, we will first explore the current challenges and existing approaches in the field of neuroscience. We will then see how nanomaterials can be utilized to address these challenges, with a focus on the distinctive properties that make them highly suitable tools for advancing neuroscience research (Fig. 1).

## Current challenges and approaches in neuroscience

The central nervous system (CNS) consists of the brain and the spinal cord. The brain primarily coordinates and processes higher-level functions (e.g. sensory processing, cognition, motor movements, etc.), while the spinal cord serves as a medium of communication between the brain and the periphery. The CNS is further composed of two key cell types: neurons and glia. Neurons serve to process changes in the environment, communicate the changes throughout the system and direct the body’s response to such changes. Glia are the supportive and most abundant cells of the nervous system which help support neighboring neurons and maintain homeostasis. The intricate network of billions of these neural cells, presumably organized in defined arrangements to impart specific neural activity, gives rise to thoughts, feelings, memories and life as we know it. In this section, we will look at three key areas of active CNS research: (a) neuro-regenerative therapies, (b) delivery of therapeutics, and (c) neuromodulation.

### Neuro-regenerative therapies

The CNS is very sensitive to damage, including infection, hypoxia, stroke, neurodegenerative diseases, and injury. The inevitable loss of neural cells makes this particularly devastating, since it leads to debilitating motor and cognitive impairment. For example, Parkinson’s disease (PD) results in the gradual loss of midbrain neurons in the *substantia nigra* which synthesize the neurotransmitter dopamine (DA), leading to rigidity and tremors [3]. Like neurodegenerative diseases, traumatic injuries can cause the loss of neural cells, in addition to complex microenvironments as the injury progresses from acute to chronic stages [4]. If kept untreated, a series of damaging conditions continue to accumulate, resulting in continued degeneration and dysfunction [5]. Due to the limited regenerative capabilities of the CNS, the loss of nervous tissue is extremely detrimental. To this end, cellular-based therapies have emerged as a promising route of therapy for CNS-related diseases and injuries [6]. The rationale is simple: replace the lost cells with new cells, in order to restore function. Cell transplantation started with clinical trials in patients with PD, in which transplantation of human fetal mesencephalic tissue rich in dopaminergic neurons was found to normalize dopamine release and reverse impairment in cortical activation [7, 8]. However, such an approach relies on the availability of donor tissue, making it an impractical long-term solution.

In this context, stem cell-based therapies have gained tremendous attention for neural disorders [9]. Stem cells are particularly suitable since they have the innate capability to self-renew, serving as a renewable source of transplantable cells that can be routinely expanded. At the same time, stem cells can differentiate into various cellular lineages, allowing for the generation of specific neural cell types of interest. Various types of stem cells, including embryonic stem cells (ESCs), induced pluripotent stem cells (iPSCs), fetal neural stem cells and mesenchymal stem cells (MSCs), have proven to be therapeutically beneficial after transplanting into damaged neural systems [10–12]. While cell transplantation initially started as an approach for cell replacement, the transplanted stem cells have been observed in recent years to imbue a number of favorable therapeutic effects in the CNS recovery process. This includes a decrease in inflammation, neuro-protection, remyelination, production of neurotrophic factors to enhance axonal regeneration and the enhancement of endogenous recovery processes [13].

Nevertheless, an active area of current research lies in achieving reproducible control of stem cell differentiation towards a pure, defined neural cell populations. The uncontrolled stem cell growth or differentiation after transplantation (e.g. teratoma formation from pluripotent cells [14]) is clearly unacceptable for clinical applications. Moreover, in vitro differentiation protocols tend to be fairly lengthy and complex; the general biologist’s approach tends to require supplementing a number of chemical compounds, biological factors or viral gene vectors, which can lead to high variability between experiments. Another common problem is the limited survival of transplanted cells and poor interaction with host tissue. For this reason, long-term viability and integration are critical factors to consider when it comes to re-establishing the damaged neuronal circuitry [13]. At the same time, the in vivo CNS microenvironment can be highly heterogeneous, with major fluctuations at the molecular and cellular level especially in the damaged site. In turn, achieving spatiotemporal control of stem cell behavior and differentiation after transplantation is quite challenging [15]. Engineering how the cell interacts with the surrounding environment is therefore critical when it comes to advancing stem cell-based neuro-regenerative therapies [16].

### Delivery of therapeutics to the CNS

Pharmacological approaches have been widely explored for the delivery of therapeutics to the CNS [17]. Therapeutic agents for CNS delivery result from screening the fundamental mechanisms of action in normal neural tissue versus diseased/damaged tissue. In this regard, therapeutics which are valuable and effective against neural disorders can come in many different forms. For instance, restoring the sufficient levels of the neurotransmitter dopamine has been reported to be a viable treatment option for patients with PD, leading to the use of dopamine precursors like levodopa or dopamine agonists [18]. On the other hand, anticancer drugs are essential for treating malignant brain tumors like glioblastoma multiforme (GBM), one of the most aggressive forms of brain cancer [19]. A number of drugs have been designed to target different molecular pathways, including paclitaxel and temozolomide [20]. Biopharmaceutics have also become attractive for CNS therapies. These include peptides, recombinant proteins, enzymes, monoclonal antibodies, and gene vectors. Compared to small molecule drugs, this class of therapeutics tends to have higher specificity and potency [21]. For instance, genomic sequencing and bioinformatics approaches have identified therapeutic targets for GBM that can be targeted with viral vectors and microRNAs [22]. In another example, in vivo administration of antibody inhibitors targeting β-secretase and α-synuclein were found to reduce amyloid-β concentrations [23] and α-synuclein aggregates [24], respectively, for treating dementia.

While multitudes of therapeutics exist for treatment, delivery to the CNS has proven to be challenging. Intracerebroventricular injection is one direct delivery option, wherein therapeutics are injected directly into the cerebral lateral ventricles [25]. However, such a strategy is highly invasive and not a feasible option for therapies requiring frequent injections. Intrathecal administration via cerebrospinal routes is also popular and generally favorable, but the restricted diffusion in the brain compared to the blood is a limiting factor [21]. These challenges arise from the fact that the CNS is highly-protected and dynamically-regulated by key physical barriers, which prevent the invasion of foreign or unwanted substances. While favorable for maintaining homeostasis, it is a critical obstacle for the systemic delivery of therapeutic agents. The blood–brain barrier (BBB) is the primary barrier protecting the CNS, consisting of a layered structure composed of endothelial cells, the capillary basement membrane, pericytes and astrocyte foot processes [26]. The tight junctions formed between the endothelial cells permits the free diffusion of small molecules, such as oxygen, carbon dioxide and water, but highly limits the movement of large molecules including most therapeutics [27].

Even though systemic delivery is limited by the BBB, targeted therapies have been developed to enhance the permeability across the BBB by: modifications of the drug, temporary disruption of the BBB using chemical or physical perturbation, catheter-based interstitial delivery or drug-eluting reservoir systems [28]. Depending on the approach, there are multiple design considerations to take into account. The first is the type of therapeutic that is to be delivered. Given that different compounds have varying chemical, physical and biological properties (e.g. small molecules versus antibody versus RNA molecule), the stability and formulation must be maintained for maximum efficacy. Second, sufficient dosing must be achieved to stay within the therapeutic window. The amount of drug administered versus the actual drug that reaches the target can significantly differ due to fluctuations in pharmacokinetics. This may thus require higher effective doses, which can be lethal, expensive, and compromise patient compliance. Balancing these considerations based on the neurological disorder to be treated is therefore essential for enhancing therapeutic delivery to the CNS.

### Neuromodulation

Mapping the neural circuitry of the brain is currently a major initiative for neuroscientists worldwide [29]. Determining the specific organization of billions of neurons, interconnected via trillions of synapses, is fundamental to unlocking how the CNS processes information to coordinate neural activity, cognition and behavior [30]. In this regard, there is a general consensus on the need for tools to better interface with the nervous system to enable the measurement and manipulation of neural signaling.

Electrodes are commonly used to record and stimulate neural activity. The most basic system is an electrolyte-filled micropipette, which is still employed for in vitro electrophysiology experiments to measure changes in current and/or potential of neurons [31]. By further modifying the physical dimensions, electrodes have been placed into mammalian brains for local neural stimulation and recording as well. One such example is deep brain stimulation, in which electrodes are implanted and stimulated near the internal globus pallidus and subthalamic nucleus to treat PD patients [32]. In order to acquire multipoint readings, microfabrication techniques and MicroElectroMechanical Systems (MEMS) have also been widely used to generate micron-scale multielectrode arrays [33]. For electrode compositions in general, a number of different metals have been explored, including gold, platinum, steel and iridium oxide [34]. However, metallic electrodes tend to be mechanically hard (50–500 GPa) compared to soft nervous tissue (0.1–1 kPa), which causes neural damage and incurs an inflammatory response after insertion [35]. Yet, electrodes must also be brought in close proximity to the target region for both effective stimulation and measurement. At the same time, long-term implantation further causes a chronic inflammatory response, leading to gliosis near the surface of the electrode and thus reducing signal transduction due to the increase in the impedance [36].

A recent technology that has significantly transformed neuromodulation approaches is optogenetics. Optogenetics involves genetically engineering cells to express photosensitive proteins, which would in turn alter their membrane potential or other cellular properties upon illumination. The core premise of this new sub-field of neuroscience lies in the selective expression of microbial opsin genes in targeted neural populations. For example, the expression of visible light-activated cation channels from algal species, such as the 470-nm blue-light responsive channelrhodopsin-2 from *Chlamydomonas reinhardtii* (ChR2) or the 535-nm green-light responsive channelrhodopsin-1 from *Volvox carteri* (VChR1), into mammalian neurons were found to transduce trains of millisecond-duration light flashes into time-locked depolarizations [37]. On the other hand, chloride-pumping halorhodopsin from *Natronomonas pharaonis* (NpHR) can hyperpolarize and thus inhibit neuronal firing using yellow-light (589 nm) [38]. Molecular engineering techniques to modify these microbial proteins and encode them in viral vectors has allowed for the introduction of these opsin genes into mammalian cells, with the first in vitro demonstration using mammalian neurons in 2005 [39]. By 2007, the first in vivo demonstration which linked optically-manipulated neural activity with specific behavioral changes in freely-moving mammals was reported [40]. By combining the spatiotemporal resolution of optical hardware with the genetic manipulation of specific cell types, optogenetics has allowed for the precise control of neural activity in select regions of the brain [41]. In other words, exposure of genetically-manipulated neuronal cells to light has facilitated the ability to modulate neural activity at a timescale relevant to brain function. Over the last decade, optogenetic techniques have elucidated neuronal circuits of numerous neural-related states and disorders including fear and anxiety, addiction, depression, reward-seeking, schizophrenia and PD [42]. While optogenetics is continuing to enable novel studies that were previously impossible, a number of fundamental limitations exist, including lack of deep tissue penetration using conventional visible light sources, the need for invasive surgeries to deliver light, and difficulties in targeting deeper brain regions [43]. A completely non-invasive approach for neural modulation would be ideal, but it may prove to be difficult due the lack of precise control in mapping or stimulating specific regions of the brain without intervention.

Regardless of the approach employed to modulate neural activity, a clear consideration for future development is to reduce invasiveness while achieving maximal quality of signal recording or stimulation. The key will be to design materials that offer optimal interfacing with intact nervous tissue, both in regard to structural (i.e. mechanical) and surface (i.e. chemical, physical) properties.

## Nanomaterials for neuroengineering

Nanomaterials have a number of unique properties that make them attractive for addressing the abovementioned challenges. For instance, the small size (below 1 micron) enables facile delivery throughout the body and into cells by crossing the plasma membrane [44]. While different cell types may have a different composition of lipids and proteins in the plasma membrane, nanomaterials cross the plasma membrane and are internalized in a size-dependent manner via endocytosis pathways, such as clathrin-mediated endocytosis, caveolae-mediated endocytosis, or phagocytosis [45]. Moreover, the surface chemistry of the nanomaterial also plays a defining role, wherein it can be adjusted to selectively bind biomolecules found on the cell membrane, in specific normal/diseased tissues, or in bodily fluids (e.g. blood, interstitial fluids, etc.). This can be achieved by conjugating cell-specific targeting ligands or antibodies to the surface. As a result, nanomaterials can be preferentially targeted to specific tissues (e.g. cancerous tissue) upon injection into the blood stream. At the same time, the nanomaterial surface can be chemically-functionalized (e.g. PEGylation) to improve circulation time in the body and evade clearance by the liver or kidney [46]. In addition, nanomaterials with a variety of different compositions, both inorganic and organic, can be synthesized. This is especially advantageous since different compositions impart specific physicochemical, thermal, electrical, magnetic, mechanical, and/or optical properties of the nanomaterial. In this section, we will explore the various types of inorganic and organic nanomaterials which have been used for to address the prominent challenges in neuroscience.

### Inorganic nanomaterials

The following inorganic nanomaterials will be described in this section: metallic nanoparticles, silica nanoparticles, magnetic nanoparticles, quantum dots and upconversion nanoparticles.

#### Metallic nanoparticles

Metallic nanoparticles are useful in medicine due their unique surface properties. Exposure to an oscillating electromagnetic field of light causes the free electrons of the metallic nanoparticle to undergo a collective coherent oscillation, termed localized surface plasmon resonance (LSPR) oscillation [47]. In turn, there is strong enhancement of the scattering and absorption cross-section at the LSPR frequency, which is advantageous since it lies in the visible spectra for noble metals such as gold (Au) and silver (Ag) [47]. This surface-based phenomenon imparts a size- and shape-dependent optical modality to AuNPs and AgNPs, which can be exploited for bio-imaging, sensing and labelling [48]. Moreover, metallic particles such as AuNPs are well-established to be biologically inert and easily amendable to functional modification, allowing for the conjugation of biomolecules such as antibodies, proteins and oligonucleotides [49].

**Fig. 2:**
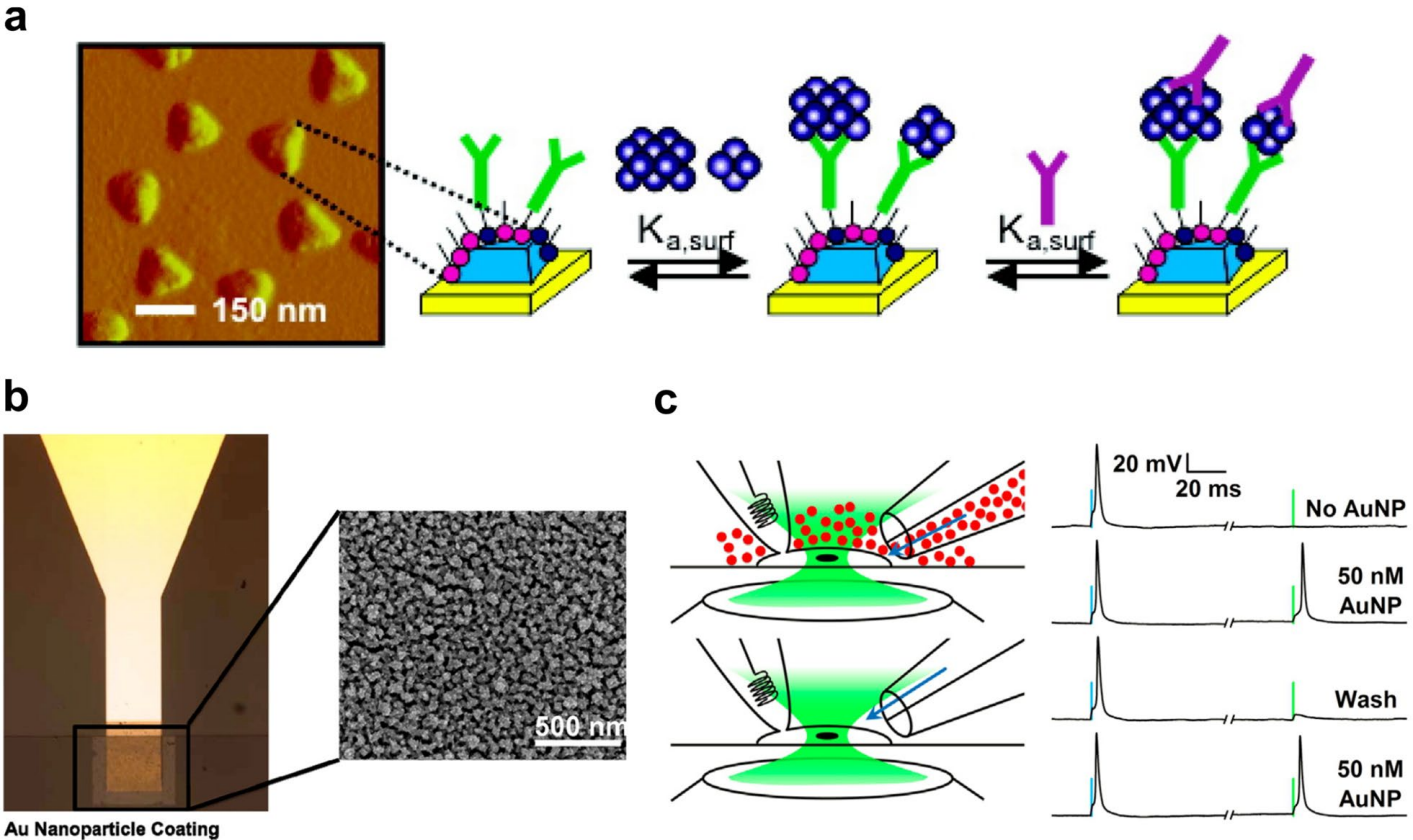
Gold nanoparticles (AuNPs). **a** Nanoscale AuNP-based optical biosensors for monitoring the interaction between amyloid-β derived diffusible ligands and specific antibodies. Reproduced with permission from Ref. [51]. Copyright (2005) American Chemical Society. **b** Layer-by-layer coating of AuNPs on electrodes for improved interfacial impedance and charge storage capacity. Reproduced with permission from Ref. [52]. Copyright (2012) American Chemical Society. **c** Schematic (*left*) for AuNPs (*red*) perfused over a patch-clamped DRG neuron through one side, then washed with fresh buffer to remove the NPs. Representative traces (*right*) of current-clamped DRG cells firing action potentials in response to current injections (*blue bars*) and 532-nm green light pulses (*green pulse*). Cells are responsive to current injection, and a bolus of AuNPs sensitized the cells to light stimulation. Reprinted from Ref. [53]. Copyright (2015), with permission from Elsevier

These properties have been exploited for numerous neural applications. In one study, 10-nm AuNPs were employed to dissolve amyloid beta (Aβ) aggregates linked with Alzheimer’s disease (AD) [50]. A specialized PEP peptide (sequence H-Leu-Pro-Phe-Phe-Asp-NH_2_) was then attached to the AuNP, which can facilitate selective binding to Aβ aggregates. Thereafter, exposure to a low gigahertz electromagnetic field prompted local heat dissipation from the AuNPs, which then dissolved the aggregates. In another study, AgNPs were employed to study the interaction of amyloid β-derived diffusible ligand (ADDL) and the anti-ADDL antibody for an optical biosensor to diagnose AD (Fig. 2a) [51].

In order to make effective electrodes, AuNPs assembled using a layer-by-layer approach to form electrodes which were shown to yield low impedance and high charge storage capacity (Fig. 2b) [52]. This initial demonstration of using AuNPs for neural interfaces showed improvements in the signal-to-noise ratio, long-term recording, and delivery of a higher charge per area of electrode to the surrounding tissue. In recent work, AuNPs have been used to further target neurons for neuromodulation [53]. Spherical 20-nm AuNPs were attached to high-avidity ligands targeting different membrane proteins of dorsal root ganglion (DRG) neurons (Fig. 2c). After binding to the neuron, the particles transduce millisecond pulses of light into heat, which sufficiently altered the membrane capacitance to elicit action potentials. This AuNP-based strategy worked well for all tested ligands to induce selective stimulation, providing an alternative to optogenetic techniques. These diverse studies highlight the potential of metallic nanoparticles for a diverse array of neuroapplications.

#### Silica nanoparticles

Silica is categorizes as a “Generally Recognized As Safe” material by the FDA, and is widely used for food additives and cosmetics [54]. Besides the favorable biocompatibility features, nanoparticles composed of silica are promising due to their robust structural stability and high drug loading [54]. It is also a highly transparent, dielectric material that does not absorb light nor conduct electrons [55]. As an inert host, silica can further serve as a matrix for the construction of well-ordered particles capable of containing small molecule drugs and biomolecules.

**Fig. 3:**
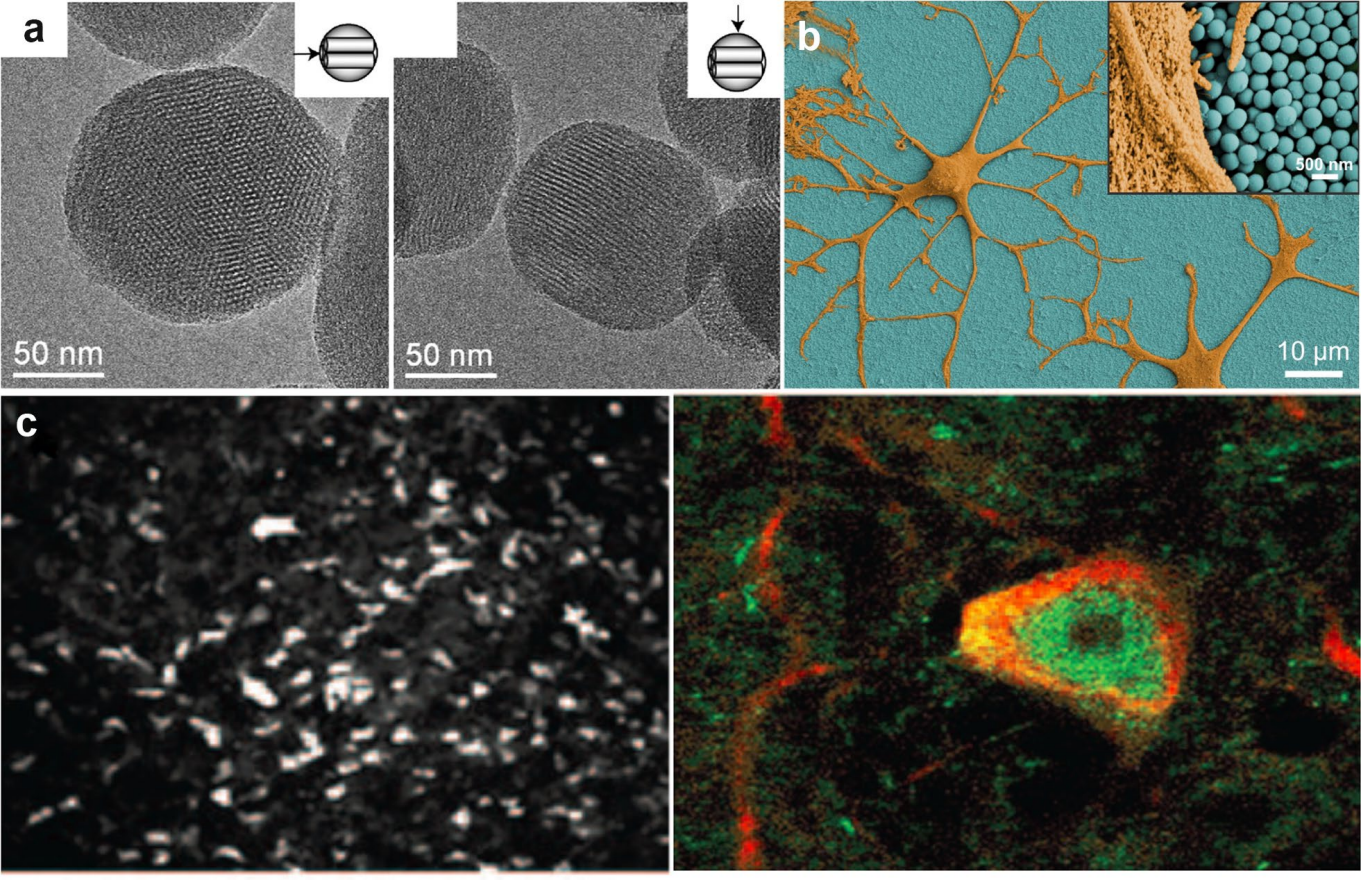
Silica nanoparticles (SiNPs). **a** Transmission electron microscopy images of mesoporous silica nanoparticles, acquired parallel (*left*) or perpendicular (*right*) to the long axis of the mesoporous channels. Reprinted from Ref. [57]. Copyright (2008), with permission from Elsevier. **b** Scanning electron microscopy image of neural stem cells (*orange*) grown on SiNP films (*blue*). Reproduced with permission from Ref. [61]. Copyright 2013 Nature Publishing Group. **c** Stereotaxic injection of SiNP-conjugated EGFP gene plasmids into mice brain. *Left side image* shows cells staining for EGFP in the *substantia nigra. Right side image* shows co-staining for transfected EGFP (*green*) and tyrosine hydrolase-positive dopaminergic neurons (*red*). Reproduced with permission from Ref. [63]. Copyright 2005 National Academy of Sciences, USA

Silica NPs are generally categorized as nonporous or mesoporous. While both are derived from an amorphous silica structure, mesoporous silica NPs have a porous structure (2–50 nm pore size), which can allow for enhanced drug loading [56]. Mesoporous silica can therefore deliver a payload (e.g. drugs, proteins, genes) by entrapping it within the pores and releasing it through passive diffusion or the controlled opening of a chemical/biological cap covering the pores (Fig. 3a) [57]. For instance, a recent study released nerve growth factors (NGF) using mesoporous silica nanoparticles, which not only prevented clearance and degradation of NGF, but improved delivery to promote nerve cell proliferation and neurite outgrowth [58]. Others have loaded agents, like ^111^ In radiolabeling, to enable multimodal in vivo imaging and tracking [59].

Recent studies have explored the response of different neural tissue-type cells, like neural stem cells, neurons, astrocytes and microglia, to silica NP treatment in order to assess optimal surface modifications that ensure minimal cytotoxicity [60]. These silica-cell interactions have further been exploited to provide nanotopographical features on interfacial surfaces. For instance, a self-assembled silica nanoparticle monolayer was employed to deliver negatively-charged RNA-based molecules (e.g. siRNA, miRNA) into neural stem cells to control neuronal differentiation (Fig. 3b) [61]. This substrate-mediated delivery for the nanoparticle film was non-toxic, highly effective, and achieved in the absence of cationic polymers.

The biocompatibility of silica has made it attractive for brain delivery. It is often used as an inert shell layer to coat other types of nanoparticles, as seen with magnetic nanoparticles delivered to track neural progenitor cells in ischemic mice [62]. Among the multitude of reports utilizing silica NPs for delivery to cells, *Bharali* et al. provided the first demonstration for in vivo delivery using silica NPs as a nonviral vector [63]. Stable aqueous dispersions of organically-modified silica NPs, bound with DNA-encoding EGFP, were prepared. Interestingly, this report described stereotaxic injection of the complexes into the mouse ventral midbrain and lateral ventricle. In addition to exhibiting no toxicity four weeks after transfection, the green fluorescence was visualized in the *substantia nigra* along with localization in tyrosine hydroxylase (TH)-positive dopaminergic neurons (Fig. 3c). This initial study gave promise for using silica NPs for in vivo delivery and brain-targeting therapies. These features of silica NPs make them an attractive option for future neural studies.

#### Magnetic nanoparticles

Magnetic nanoparticles (MNPs) are attractive due to the superior contrast enhancement they offer for in vivo imaging. Magnetic resonance imaging (MRI) is one of the most widely used medical imaging techniques, which relies on measuring the relaxation times of excitable hydrogens in the tissue to acquire high-resolution images. Since such intrinsic differences tend to be insufficient for obtaining a detectable signal, contrast agents bearing paramagnetic or superparamagnetic properties are often used. MNPs, such as iron oxide-based particles (Fe_2_O_3_ and Fe_3_O_4_), are excellent MRI contrast agents for improved sensitivity in *T*-_2_-weighted imaging [64]. MNPs composed of iron oxide are clinically approved as MRI contrast agents, in which Feridex and Resovist well-known commercial products [65].

MNPs tend to be most effective when the size is typically around 10–20 nm [66]. Over the years, synthetic procedures have been optimized to: (a) facilitate the incorporation of metals into the magnetic core that offer enhanced magnetic properties (e.g. zinc, cobalt, nickel), (b) coat with organic species (e.g. surfactants, polymers) to prevent degradation, and (c) deposit inorganic shell layers (e.g. silica, gold) for greater stability and additional surface functionalization [66].

The inherent magnetic properties of MNPs enable these particles to serve as useful multifunctional neural platforms. For instance, MNPs have been designed to selectively cross the BBB by minimizing the size and coating the surface with biocompatible polymer layers and chemical functionalities [67]. Further complexing with tumor-specific peptides (e.g. chlorotoxin, CTX) allowed the MNPs to be targeted to highly invasive glioma brain tumors in mice models [67]. Moreover, this entire process could be monitored using MRI, making MNPs a versatile nanoparticle platform.

**Fig. 4:**
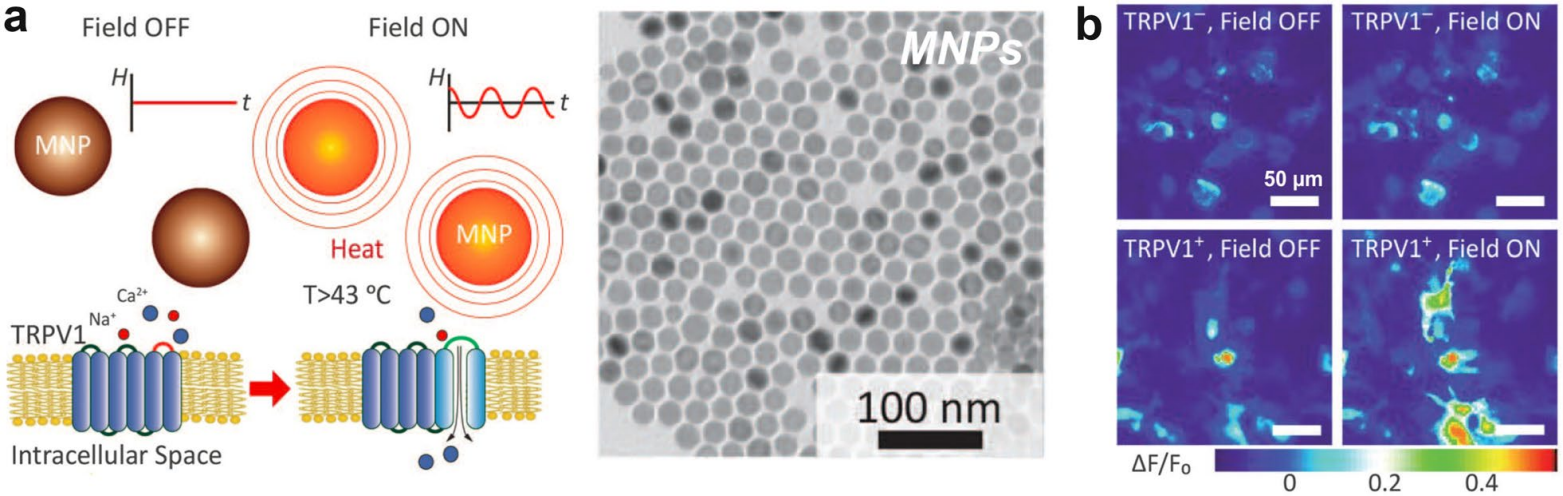
Magnetic Nanoparticles (MNPs). **a** Experimental scheme (*left*) showing magnetothermal deep brain stimulation using MNPs to open temperature-sensitive TRPV ion channels. Transmission electron microscopy image (*right*) showing the size and distribution of the MNPs. **b** *Color maps* of fluorescence intensity changes for TRPV1− and TRPV1+ cells before and during magnetic field exposure in the presence of MNPs. From Ref. [71]. Reprinted with permission from AAAS

The high saturation magnetization properties of MNPs has also facilitated magnetic-based targeting. In this case, the dragging force of a permanent magnet is used to deliver MNPs to a target site. This has been demonstrated for both in vitro gene delivery to neural stem cells [68] and in vivo for drug/gene delivery to brain tumors of 9L-gliosarcoma-bearing rats [69]. Along with magnetic targeting, MNPs are becoming attractive for neuromodulation as well. In one study, 30-nm CoFe_2_O_4_-BaTiO_3_ nanoparticles were injected through the mouse’s tail vein and forced to cross the BBB via a d.c. field, and then used to modulate the electric waveforms in the brain upon exposure to an external a.c. field [70]. In another recent demonstration, MNPs were even used to stimulate deep brain structures in vivo through magnetic heating (Fig. 4a) [71]. The heat-sensitive capsaicin receptor TRPV1 was expressed with lentiviruses in the ventral tegmental area (VTA), followed by MNP injection into the same region. Subsequent exposure of the mice to alternating magnetic fields induced heat dissipation by hysteresis from the MNPs, permitting neuronal excitation by TRPV1-activation for up to one month after MNP injection (Fig. 4b). These diverse properties of MNPs offer unique applications for neural research.

#### Quantum dots

Quantum dots (QDs), also referred to as semiconductor nanocrystals, have emerged as an exciting class of fluorescent probes. Conventional organic dyes readily photobleach upon prolonged irradiation, and tuning their optical properties is a general synthetic challenge [72]. In contrast, QDs are resistant to photobleaching, extremely bright, and exhibit size-dependent emission wavelengths. In particular, QDs tend to have a broad absorption spectrum and a narrow emission spectrum. This allows for the generation of QDs that can be excited with a single wavelength yet emit at different wavelengths [73]. Along with the small size range (~ 2-10 nm), this enables unique applications of QDs in multiplex imaging and biosensing.

Early on, a major limitation of QDs was the possibility of cytotoxic side effects. The most prominent QDs are composed of heavy metal ions, such as cadmium, selenide and tellurium, which exhibit adverse effects upon exposure to cells and tissues [74]. Strategies have been devised to overcome these issues, which include coating the core QD with inert shells (e.g. ZnS, silica) to prevent leaching of toxic elements [75], or using biocompatible elements to generate non-toxic QDs (e.g. CuInS_2_, ZnS-AgInS_2_) [76].

These synthetic optimizations have significantly improved QDs and made them attractive for neuroapplications. An early study used different surface functionalization strategies to complex biomolecules like siRNA to QDs for the delivery into U87-glioblastoma brain tumor cells [77]. In such a case, the innate fluorescence property allows QDs to serve as a single vehicle for drug/biomolecule delivery, visualization and monitoring. For instance, the QDs can be targeted to specific cell types to enable cellular tracking within the body. A recent study showed that the conjugation of QDs with cell-penetrating lipopeptides and the subsequent injection into intact embryonic chick brains helped to identify and monitor neural stem cells as they migrate in the developing brain [78].

The long-term stability and robust fluorescence properties of QDs make them useful for mechanistic studies as well. For instance, the movement of QD-labeled nerve growth factor (NGF) was tracked in cultures of rat dorsal root ganglion (DRG) neurons to understand the mechanism of retrograde axonal transport [79]. Preliminary studies have also exploited the optoelectronic properties of QDs to activate ion channels on neurons [80]. In this case, remote-controlled membrane depolarization or hyperpolarization was achieved in cortical neurons by activating K^+^ and Na^+^ channels using QD films and QD-coated micropipettes. The combination of the innate optical properties with the additional bioconjugation capabilities make QDs a favorable option for advanced studies.

Carbon-based quantum dots (CQDs or C-dots) have also recently emerged as a new category of fluorescent probes, with sizes below 10 nm. CQDs were first discovered as a major impurity during the purification of single-walled carbon nanotubes using preparative electrophoresis [81]. Thereafter, a number of synthetic strategies were devised to generate CQDs, including electrochemical carbonization, laser ablation and hydrothermal treatment [82]. Compared to the traditional semiconductor-based quantum dots described above, CQDs have enhanced photoluminescence, solubility in water and biocompatibility [82]. These remarkable properties have made CQDs particularly useful for applications in bioimaging and biosensing [83]. In the context of neuroscience, CQDs were recently employed to target brain cancer glioma in mice [84]. Synthesized using a simple thermolysis route with d-glucose and l-aspartic acid as starting materials, the as-prepared CQDs not only showed tunable emission spectra, but also intrinsic targeting to brain C6 glioma cells. While there are still limited investigations using CQDs for neuroapplications, such favorable properties makes this class of QDs attractive for future studies.

#### Upconversion nanoparticles

**Fig. 5:**
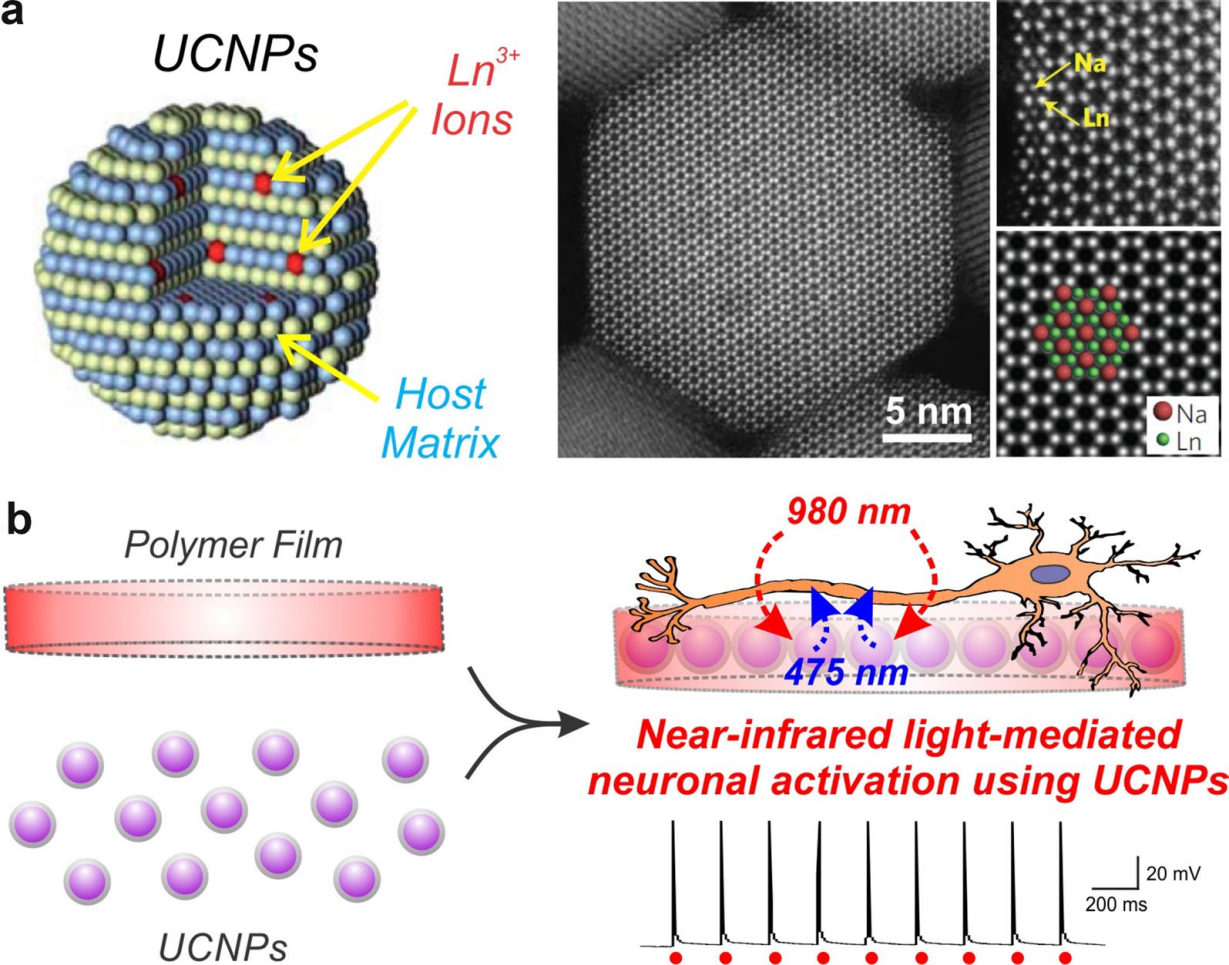
Upconversion nanoparticles (UCNPs). **a** Schematic illustration (*left*) of UCNPs composed of a crystalline host and lanthanide dopant ions embedded in the host lattice. High-resolution transmission electron microscope image (*center*) revealing the single-crystalline nature of the crystal. Enlarged view (*right*) shows lanthanide (Ln) and sodium host (Na) atomic columns. Reproduced with permission from Ref. [87]. Copyright (2011), Nature Publishing Group. **b** Embedded within polymeric films, UCNPs can serve as internally excitable light sources that convert NIR light into blue light, thus facilitating optogenetic activation of channelrhodopsin (ChR)-expressing neurons. Reproduced form Ref. [92] with permission from The Royal Society of Chemistry

Upconversion nanoparticles (UCNPs) have attracted significant biomedical interest due to their ability to absorb low-energy photons and emit high-energy photons. In other words, UCNPs convert long-wavelength near-infrared light (NIR; >800 nm) to short-wavelength visible light (300–700 nm), known as an anti-Stokes process [85]. This phenomenon is possible due to the unique optical properties of the lanthanide-series elements, which are used as dopants within a crystalline host matrix, such as NaYF_4_ (Fig. 5a). Two different types of lanthanide-dopants are usually employed: a sensitizer and an activator. Upon NIR irradiation, the sensitizer (e.g. Yb^3+^) harvests NIR energy and transfers it through a non-radiative process to the activator (e.g. Er^3+^, Tm^3+^). Further transitions within the activator to higher energy levels then occur until radiative emission occurs [86].

The ladder-like arrangements of energy levels in trivalent lanthanide ions (Ln^3+^) thus allows for visible light emissions through various energy transfer pathways, depending on the pre-selected ion pairing [87]. As a result, while conventional organic dyes are sensitive to their chemical surroundings, the shielded 4f–4f intra-configurational transitions in UCNPs permit emissions that are independent of the particle size and environment [86]. The fixed energy levels, high resistance to photoblinking and photobleaching, long luminescence lifetimes (micro/milli-second) and the upconversion process using deep-penetrating NIR light makes UCNPs ideal as in vivo probes [88].

UCNPs first emerged in neuroscience research for imaging and cancer application. In one study, the conjugation of the RGD peptide to the surface of NaYF_4_:Yb^3+^/Tm^3+^ UCNPs allowed for targeted imaging of nude mice bearing human glioblastoma U87MG tumors [89]. Others have conjugated neurotoxins such as chlorotoxin, which has the ability to target primary brain tumors, for imaging of xenograft glioma tumors in Balb-c nude mice in vivo and ex vivo [90]. In a recent study, a UCNP-based sensor was also designed for the detection of Zn^2+^ in brain slices from mice bearing Alzheimer’s disease [91].

The application of UCNPs has recently been extended to optogenetically modulate neuronal activity using near-infrared light. Optogenetic approaches are contingent upon delivering a sufficient dose of visible light to the target opsin-expressing cells. Since visible light is highly scattered in tissue, invasive procedures are required to precisely implant optical fibers at the target site. In an alternative strategy, UCNPs have been shown for the first time to serve as mediators for converting deep-penetrating NIR light into visible light (e.g. blue light) to facilitate optogenetic neuronal control [92]. Embedding the UCNPs in a biodegradable polymer can further ensure a sufficient neural interface for repeated NIR-stimulation (Fig. 5b). There is continued efforts to enhance the upconversion efficiency in order to improve UCNP-based optogenetic control [93], as well as open new applications of UCNPs for neural research.

### Organic nanomaterials

The following organic nanomaterials will be discussed in this section: liposomes/micelles, dendrimers, polymeric NPs and carbon-based nanomaterials.

#### Liposomes/micelles

**Fig. 6:**
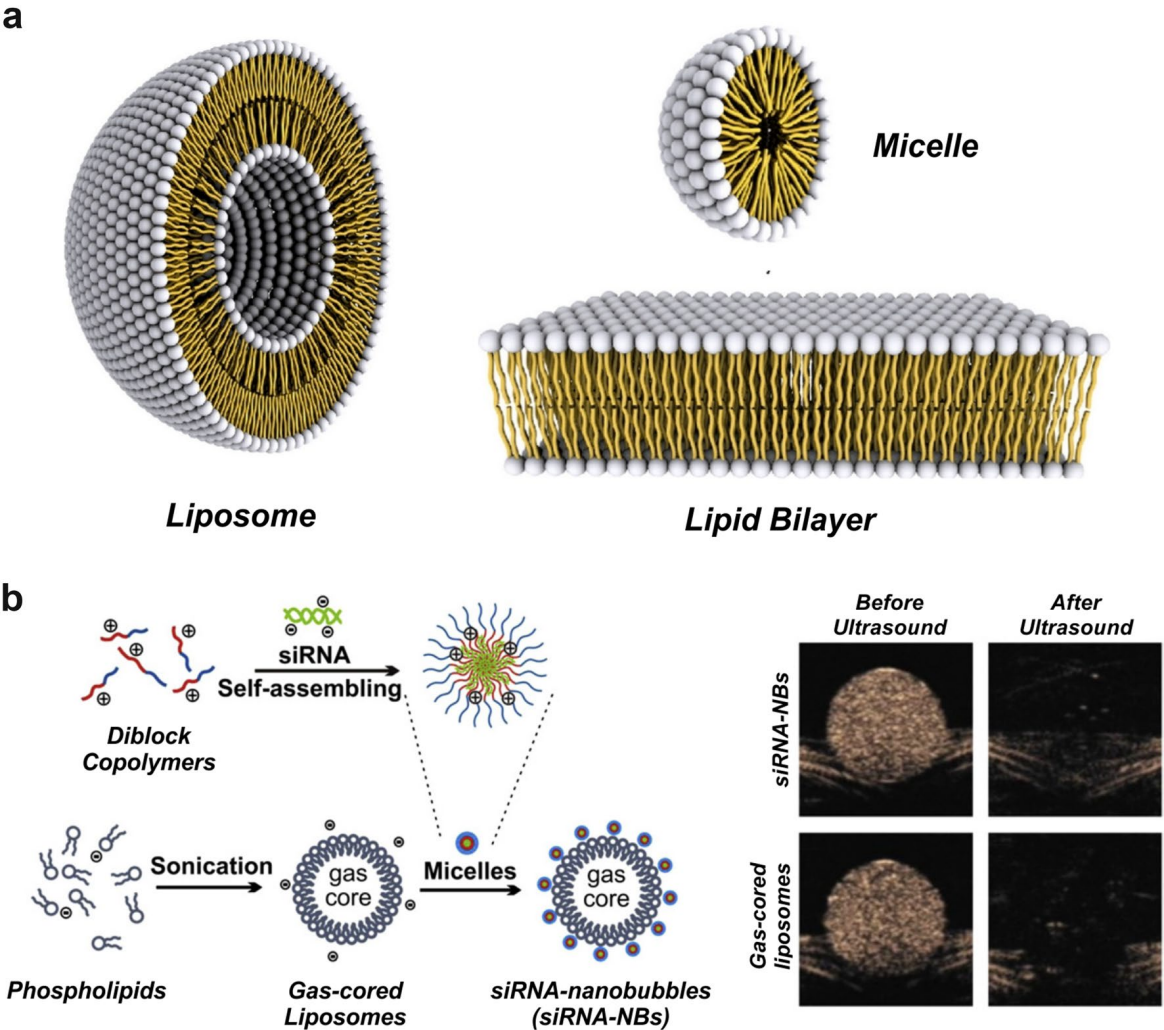
Liposomes and micelles. **a** Amphiphiles can assemble into liposomes (*left*), micelles (*top*) or lipid bilayers (*bottom*). Reproduced with permission from Ref. [95]. **b** Schematic (*left*) for preparation of siRNA-nanobubles (siRNA-NBs) using positively-charged siRNA micelles and gas-cored liposomes. Contrast enhanced ultrasound images on the right show the dispersion of siRNA-NBs and gas-cored liposomes (indicated by diminution in *gray-scale* intensity) upon low-frequency ultrasound exposure. Reprinted from Ref. [109]. Copyright (2013), with permission from Elsevier

Amphiphiles, which contain both hydrophilic and hydrophobic domains, are powerful building blocks in biology [94]. They self-assemble in order to minimize the energetically unfavorable interaction of hydrophobic moieties with the surrounding water molecules, leading to the formation of well-defined nanoassemblies [95]. An example of this phenomena is the cellular membrane, which is the dynamic assembly of phospholipids. The chemical control of this assembly process has enabled the generation of various types of amphiphilic nanocarriers, in which liposomes and micelles are two well-known categories (Fig. 6a) [94].

Liposomes are spherical-shaped vesicles comprised of one or more vesicular bilayers (lamellae) [96]. This unique bilayer formation into a spherical assembly generally results in a core of aqueous solution, which can be loaded with hydrophilic compounds. At the same time, hydrophobic compounds can be loaded within the lipid bilayer itself. Liposomes are usually composed of natural phospholipids such as sphingomyelin and glycerophospholipids, or synthetic polymers such as block copolymers [97]. Micelles, on the other hand, are spherical-shaped assemblies that have a hydrophilic exterior and a hydrophobic interior [98]. In contrast to molecules used to form liposomes, micelles consist of amphiphilic molecules (also known as surfactants) with one hydrophobic tail linked to one hydrophilic head. In turn, dispersion in water leads to the spontaneous formation of micelles, wherein the tail portion of the molecules sequester away from the water molecules into a highly hydrophobic core [99]. Pluronic block copolymers, such as ethylene oxide and propylene oxide, are wide-used for micelle formation [100].

Amphiphilic-based nanoassemblies like liposomes and micelles have been utilized for several decades now in neuroscience research, particularly for the gene/drug delivery to the CNS. Early work demonstrated liposome-based drug vehicles capable of delivering anticancer agents like daunomycin across the BBB into the rat brain [101]. In such cases, modification with targeting moieties (e.g. antibodies) and stabilization with polyethylene glycol (PEG) conjugation to increase in vivo circulation times proved to be essential. Recent studies have advanced the application of multifunctional liposomes to address a variety of neurological ailments in vivo, including neuroprotection after cerebral ischemia [102], targeting gliomas [103], treating brain metastasis [104] and reducing β-amyloid plaques in Alzheimer’s disease [105]. Polymeric micelles have been similarly applied. In one study, the cell-penetrating peptide TAT was anchored to micelles in order to deliver the antibiotic ciprofloxacin across the BBB to treat brain infections [106].

These amphiphilic nanoassemblies have also grown popular for other specialized neural applications. For example, liposomes have been used to mediate neural regeneration, wherein genes coding for neurotrophic growth factors (e.g. GDNF, NGF) were delivered after neuronal injury [107, 108]. The in vivo expression of these growth factors was seen to promote axonal regeneration and improve locomotive function in adult rats [107]. Besides serving as a drug/gene carrier, liposomes and micelles have been uniquely used as externally-triggerable agents. For example, a hetero-assembly of siRNA-complexed polymeric micelles and gas-cored liposomes was recently synthesized to develop an ultrasound-based nanobubble therapy (Fig. 6b) [109]. In this case, the ultrasound-sensitive gas-cored liposomes carried the siRNA-loaded micelles, resulting in enhanced delivery efficiency and gene silencing in a mouse glioma model. In general, the biocompatibility, facile surface functionalization, and lack of immune response have made these amphiphile-based nanocarriers invaluable.

#### Dendrimers

**Fig. 7:**
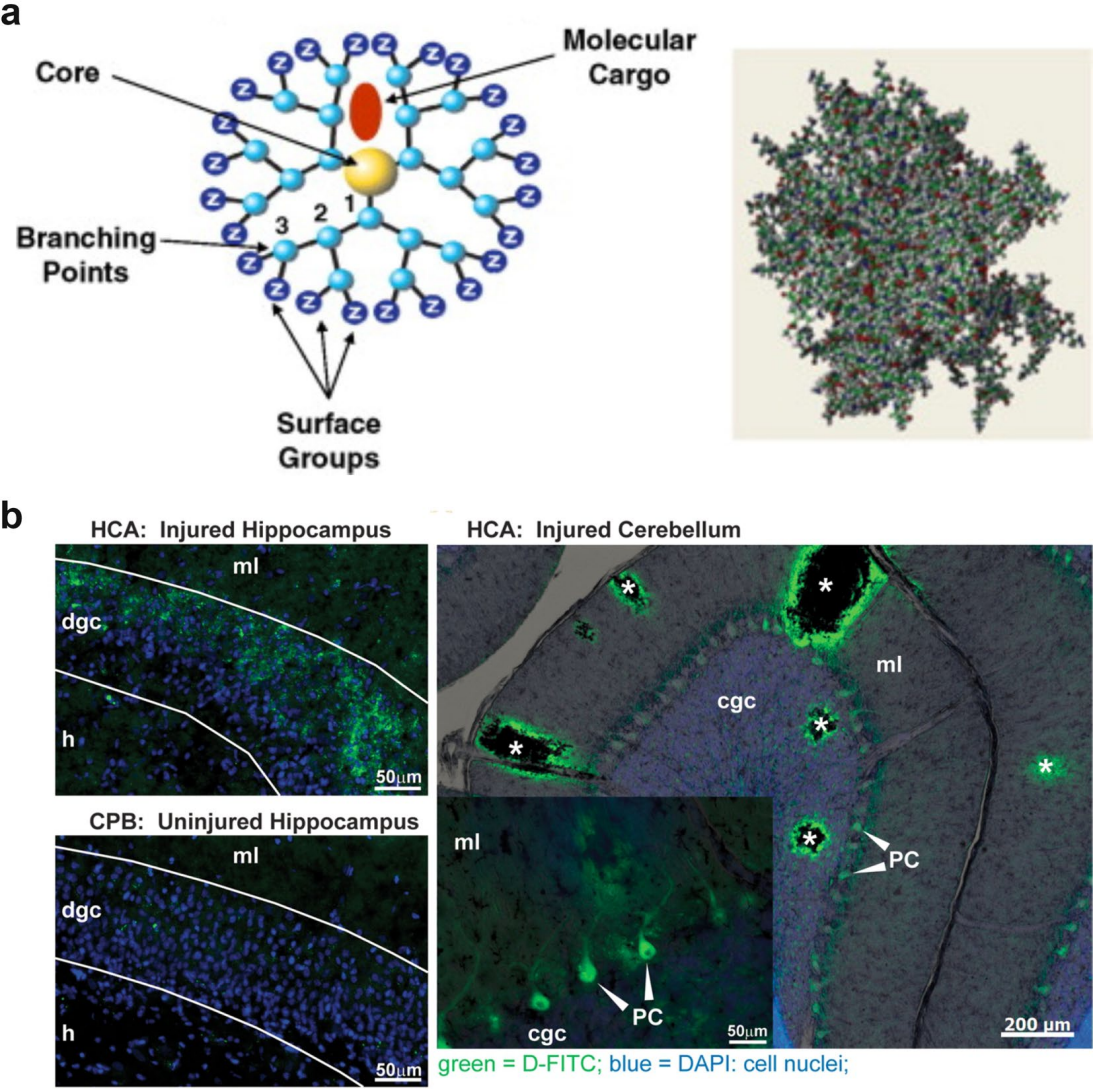
Dendrimers. **a** Schematic 2D representation (*left*) of a dendrimer containing three generations (branching points) as indicated by* numbers*. A 3D representation (*right*) of dendrimer G3 showing space-filling structure. Reprinted form Ref. [111]. Copyright (2009), with permission from Elsevier. **b** Systemically administered dendrimer-FITC (D-FITC) localize in the injured brain. D-FITC was administered IV after 2 h hypothermic circulatory arrest (HCA) or cardiopulmonary bypass (CPB); fluorescent labeling was examined 48 h later. D-FITC labeling in the outer part of the hippocampal dentate granule cell layer (dgc) after HCA closely corresponds to the distribution of apoptotic nuclei detected with DAPI (*top*). Negligible labeling is observed in the hippocampus after CPB, which causes little to no injury (*bottom*). In cerebellum after HCA, D-FITC is prominent in many Purkinje cells (PC), which receive dense glutamatergic input and are often injured, and surrounding small hemorrhages (*). Reproduced with permission from Ref. [117]. Copyright (2014) American Chemical Society

Dendrimers are synthesized by the cross-linking of repeating monomer subunits [110]. This regular arrangement of the monomers results in a highly-branched and well-defined hierarchical structure. Emanating from an initiator core, the layer-by-layer expansive growth allows for the synthesis of varying ‘generations’ of dendrimers with different hydrodynamic sizes, branching points and surface functionality (Fig. 7a) [111]. Further modification of the surface to introduce chemical functionalities (e.g. positive-charged amine groups) can render dendrimers useful for complexation with drugs and gene vectors [112, 113]. Various types of dendrimer systems have been used for biological studies, including poly(propylene imine) (PPI) and poly(amidoamine) (PAMAM) [111].

Dendrimers are promising for targeted delivery to the brain. Attaching different classes of drugs for CNS therapies, including anticancer, anti-inflammatory, and antimicrobial agents, is facilitated by either encapsulation within the dendrimer or through chemical bonding [114]. Studies have shown the hydroxy-functionalized PAMAM dendrimers to be non-toxic, yet only minimal uptake was observed in both healthy and tumor-bearing animals [115]. However, enhanced PAMAM dendrimer uptake was observed into the brain following neuroinflammation, possibly due to impairment in the BBB [116]. The localization of dendrimers to activated microglia after systemic administration was observed in rabbit models of cerebral palsy, which in turn allowed for the targeted delivery of N-acetyl-l-cysteine (NAC), an antioxidant and anti-inflammatory agent [116]. Recent work further advanced these findings from the small animal rabbit injury model to a larger canine model [117]. After systemic administration, PAMAM localized to the injured neurons and microglia in the brain of canines (Fig. 7b), allowing for the delivery of both NAC and valproic acid for enhanced neuroprotection [117]. Such seemingly inherent targeting, in addition to further modification of the dendrimer to incorporate therapeutics, makes the dendrimer class of nanomaterials clinically-relevant.

#### Polymeric nanoparticles

Polymeric nanoparticles are composed of natural or synthetic polymers, and are generally biodegradable. Examples of synthetic polymer-based nanoparticles include poly(lactide-co-glycolide) (PLGA), poly(butylcyano-acrylate) (PBCA), poly(glycolic acid) (PGA) and poly(lactic acid) (PLA), and natural polymers include alginate, collagen and gelatin [118]. Ranging in size from 10 nm to upwards of several micrometers, these carriers can be formed into solid nanospheres (matrix-based) or nanocapsules (liquid core surrounded by polymer shell). These carriers can further contain therapeutics by: (a) dissolving, absorbing or dispersing throughout the matrix; (b) covalent attachment to the polymer matrix; or (c) encapsulation within the core [119]. The key advantage of using a polymeric-based particle is that it provides a protective coating for the therapeutic, which ensures enhanced stability and efficacy after in vivo administration compared to the free form [119]. Moreover, the pharmacokinetic properties of the therapeutic-loaded polymeric nanoparticles can be further enhanced by functionalizing the surface, which can enable targeted delivery as well as increased permeability through the BBB [120].

The CNS delivery of drugs or biomolecules is a key application of polymeric nanoparticles. Early studies found PBCA nanoparticles to be effective for CNS delivery. In one study, the antinociceptive opioid hexapeptide dalargin was transported across the BBB, which otherwise could not enter the brain in sufficient quantities to induce antinociception [121]. Follow-up studies also showed the BBB translocation of such acrylate-based particles by surface conjugation of apolipoproteins [122]. Nonetheless, the rapid degradation of PBCA serves to minimize cytotoxicity resulting from polymer accumulation, and further permits the delivery of a variety of drugs to the CNS, including doxorubicin, methotrexate, loperamide and temozolomide [123].

Polyester-based nanoparticles like PLGA have been observed to be safer alternatives for brain delivery since the degradation products are mainly water and carbon dioxide [124]. Besides drugs, polymeric nanoparticles have been useful for the sustained release of growth factors to treat neurodegenerative disorders. In a Huntington’s disease rat model, the local administration of nerve growth factor (NGF)-loaded PLGA enabled neuroprotection after excitotoxin quinolinic acid injections [125]. Similar loading with other tropic factors and neurotransmitters has led to significant results for neuroprotection and repair [18, 126]. Polymeric nanoparticles have also been used to direct neural stem cell behavior in vivo. For example, PEI-based nanoparticles were complexed with retinoic acid to control neural differentiation in the subventricular zone (neural stem cell niche) [127] and after ischemia [128]. In this way, polymeric nanoparticles will continue to provide utility in advancing studies.

#### Carbon-based nanomaterials

**Fig. 8:**
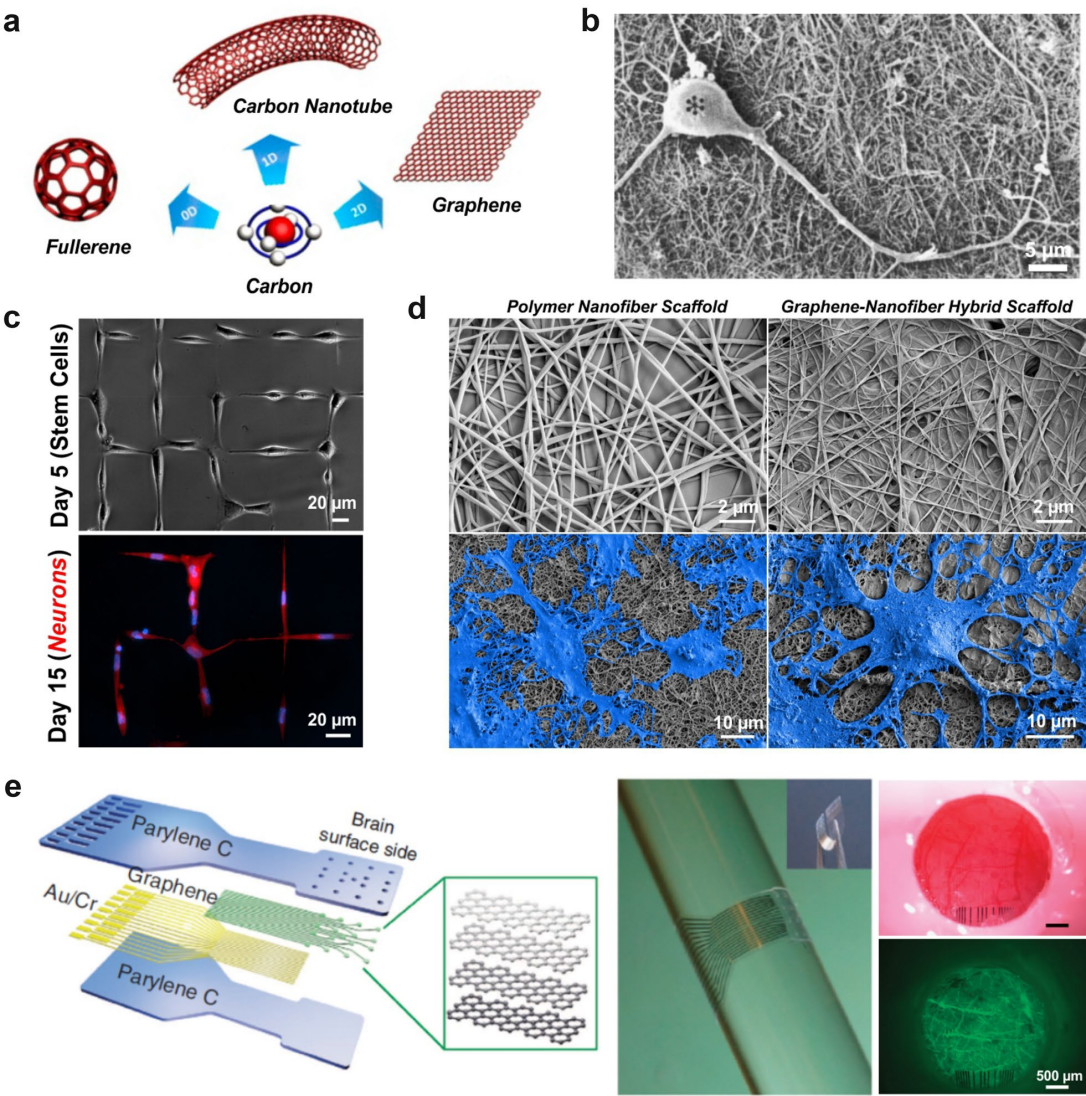
Carbon-based nanomaterials. **a** Carbon nanomaterials include zero-dimensional fullerenes, one-dimensional carbon nanotubes (CNTs), and two-dimensional graphene. Reproduced with permission from Ref. [129]. **b** Scanning electron microscopy image of embryonic hippocampal neurons growing on dispersed multiwall CNTs. Reproduced with permission from [137]. Copyright (2000), Springer. **c** Adipose-derived stem cells grown on graphene patterns show enhanced differentiation into neuronal cell fate. Reprinted with permission from Ref. [146]. Copyright (2015) American Chemical Society. **d** Graphene-coating on polymeric nanofibers promoted the selective differentiation of neural stem cells into oligodendrocytes. Reproduced with permission from Ref. [147]. Copyright 2014, WILEY–VCH Verlag GmbH & Co. KGaA, Weinheim. **e** Diagram (*left*) of graphene-based carbon-layered electrode array device construction showing the layered structures. Demonstration of the flexibility of the device (*center*) by wrapping around a glass bar with a radius of 2.9 mm. Bright-field image (*right*, *top*) and fluorescence image (*right*, *bottom*) of the device implanted on the cerebral cortex of a mouse beneath a cranial window. Green labelling indicates the vasculature. Reproduced with permission from Ref. [152]. Copyright (2014), Nature Publishing Group

Carbon-based nanomaterials are becoming attractive due to their unique optical, thermal, mechanical, electrical and chemical properties. Composed of sp^2^-bonded graphitic carbon, these nanomaterials are categorized into zero-dimensional, one-dimensional and two-dimensional structures (Fig. 8a) [129]. Laser ablation of graphite was used to isolate the well-known C_60_ buckyball in 1985, a zero-dimensional fullerene derivative, which was the first carbon nanomaterial to be isolated [130]. Soon after, one-dimensional carbon nanotubes (CNTs) were prepared using arc discharge techniques in 1991 [131]. The cylindrical carbon structure has an extended sp^2^ carbon with physical properties that can be tuned, such as the diameter, length, number of walls/cylindrical layers and chirality [132]. CNTs are in fact made of graphene sheets wrapped onto themselves, but two-dimensional graphene was not isolated until 2004, using mechanical exfoliation [133]. As a single-atom thick sheet, graphene exhibits a number of remarkable properties, including: high planar surface area, superior mechanical strength, unparalleled thermal conductivity, and favorable electronic properties and optical properties [134]. Further modifications to the graphene surface, like oxidation to create graphene oxide, has resulted in derivatives with complementary properties for biological studies (e.g. enhanced water solubility, facile functionalization for biomolecule conjugation, etc.) [135].

Besides serving as a remarkable material for the biomedical field in general, carbon-based nanomaterials have become especially useful in neuroengineering, where the focus is the development of devices or surfaces to effectively interface with the CNS [136]. The first report using these materials for neural research was the growth of embryonic rat brain neurons on multi-walled CNTs (Fig. 8b) [137]. This early work highlighted the importance of modifying the CNT surface for enhanced neurite outgrowth, a critical feature for enhanced in vivo performance in terms of biocompatibility, neuron growth and neurite/axonal elongation. While graphene is similar to CNTs in many ways, the two-dimensional structure and flexibility of graphene allows for facile coating on numerous types of cell culture surfaces. The early demonstration of the biocompatible interaction of neurons with graphene showed favorable long-term outcomes, in which mouse hippocampal neurons had enhanced neurite sprouting and outgrowth on graphene-coated tissue culture polystyrene (TCPS) compared to bare TCPS substrates [138].

The promising results from neuronal cultures led to the examination of carbon-based nanomaterials for stem cell cultures. One of the earliest studies showed the successful differentiation of mouse NSCs on single-walled CNT-polyelectrolyte multilayer thin films into neural cells [139]. The viability, neurite outgrowth and neural marker expression was found to be comparable between the conventional poly-l-ornithine (PLO) surface and the CNT surface. Further modifications of CNTs has also resulted in enhanced neuronal differentiation, both by using different types of stem cells, like hESCs [140] and hMSCs [141], and by combining with other biomaterials, like collagen [142] and silk [143]. Graphene-coated surfaces have also shown similar enhancements in neuronal formation [144], axonal alignment [145], neuronal patterning [146] and oligodendrocyte differentiation [147] (Fig. 8c, d).

An exceptional feature of carbon-based nanomaterial for neural research is its inherent electrical properties [148]. For example, the large electrical conductivity originating from the highly-mobile π-electrons has been seen to influence neurite outgrowth of dissociated hippocampal neurons grown on single-walled CNT films [149]. Combining graphene with polyethylene terephthalate (PET), the hybrid was shown to act as an electrical stimulator, in which electric field stimulation caused increased cell-to-cell couplings in a human neuroblastoma cell line [150]. Interestingly, this electrical field stimulation was achieved in a non-contact manner, and was non-cytotoxic. Implanting electrodes coated with carbon nanomaterials has also shown great promise. In one study, CNT coating on conventional tungsten and stainless steel wire electrodes was seen to decrease electrode impedance and increase charge transfer, permitting both enhanced recording and electrical stimulation in rat and monkey brains [151]. Recently, graphene-coating of microelectrode arrays has even enabled in vivo imaging, neurophysiological recording and optogenetic activation in the rodent brain (Fig. 8e) [152]. Considering these exceptional features, carbon-based nanomaterials have great promise for improving neural interfaces. With more and more studies verifying the compatibility of such materials, there is immense scope for using these robust materials in translational studies.

## Conclusions

From both a fundamental and an applied science point-of-view, nanotechnology and nanoscience has greatly advanced in a relatively short period of time. Nanomedicine in particular has seen a steady progress in the last two decades, with tremendous efforts being placed in translating these advances to the field of neuroscience. A wide array of nanomaterials show promise for enhancing our understanding of the CNS, moreover offering therapeutic opportunities in CNS-related treatment. Many of the inorganic-based nanomaterials, such as metallic nanoparticles, magnetic nanoparticles and quantum dots, are being extensively employed as imaging agents. Other inorganic nanomaterials provide unique advantages, such as enhanced small molecule/biomolecule loading with silica nanoparticles and improved optical penetration with upconversion nanoparticles. This makes them quite versatile and adaptable to crucial neuroapplications such as CNS drug delivery and deep tissue imaging, respectively. In contrast, organic nanomaterials such as micelles, liposomes dendrimers and polymeric nanoparticles are generally biocompatible and biodegradable right from the start. In addition, carbon-based nanomaterials offer superior material properties, making this class of nanomaterials attractive candidates for neural interfaces. The availability of such a diverse nano-toolkit has changed the way scientists approach challenges in neuroscience.

Nevertheless, a growing need exists to create nano-based platforms that bear multiple functionalities on a single platform. This is mainly due to highly complex nature of the CNS, and furthermore it’s sensitivity to slight damage and the consequent limited capability for autonomous repair. As a result, approaches that enable maximal effectiveness with minimal perturbation of the intact tissue would be ideal. In developing the next generation of nanoscale CNS platforms, critical design criteria consist of: attachment of multiple types of therapeutic agents, spatiotemporal control within the body, built-in modalities for long-term tracking, and capabilities to record and modulate neural activity. Integrating these features on a single nanoplatform holds remarkable potential for utilizing the nanomaterial toolkit for advanced neuroengineering applications.
